# Lack of conservation of bacterial type promoters in plastids of Streptophyta

**DOI:** 10.1186/1745-6150-5-34

**Published:** 2010-05-10

**Authors:** Vassily A Lyubetsky, Lev I Rubanov, Alexandr V Seliverstov

**Affiliations:** 1 Institute for Information Transmission Problems of the Russian Academy of Sciences, 19, Bolshoy Karetny per., Moscow, 127994, Russia

## Abstract

We demonstrate the scarcity of conserved bacterial-type promoters in plastids of Streptophyta and report widely conserved promoters only for genes *psaA, psbA, psbB, psbE, rbcL*. Among the reasonable explanations are: evolutionary changes of sigma subunit paralogs and phage-type RNA polymerases possibly entailing the loss of corresponding nuclear genes, *de novo *emergence of the promoters, their loss together with plastome genes; functional substitution of the promoter boxes by transcription activation factor binding sites.

**Reviewers:**

This article was reviewed by Dr. Arcady Mushegian, and by Dr. Alexander Bolshoy and Dr. Yuri Wolf (both nominated by Dr. Purificación López-García).

## Background

Genes evolve at different rates. Various hypotheses try to explain, or at least to correlate, the evolutionary rate (sequence conservation) and the functional properties of the protein-coding gene. As far as we know, there is no published evidence on searching for the plastid promoters at the genome scale. This problem should probably be addressed separately for nuclear, plastome and mitochondrial genomes, different taxonomic lineages and different RNA polymerase types. In particular, multisubunit RNA polymerase (PEP), which has the core enzyme encoded in plastome and the sigma subunit in nucleome, binds bacterial type promoters (PEP-promoters); and monosubunit RNA polymerases (NEP), which is nucleome-encoded, binds NEP-promoters. Here we report a study of PEP-promoters of plastome genes in representatives of the green line (Viridiplantae, including Chlorophyta and Streptophyta; Euglenozoa, Rhizaria, in particular Cercozoa; Glaucocystophyceae) and the red line (Rhodophyta, stramenopiles, including Bacillariophyta, Pelagophyceae, Raphidophyceae, Xanthophyceae; Cryptophyta, Haptophyceae, Apicomplexa). Add. file 1 describes the complete list of studied species with plastids, organized according to the NCBI Taxonomy. Plastid genes are believed to be evolutionarily conserved across large taxonomic lineages [[1], section 9.7c], although the authors are unaware of systematic studies on their promoters conservation. Instead, there is ample published research on the promoter comparisons within small lineages, largely the studies of the promoters and their transcription factors in gamma- and alpha-proteobacteria [2]. Further, some pairs of closely related species have been shown to possess largely diverged promoters [3,4]. We have reported an evolutionary labile promoter for the *ndhF *gene in a narrow lineage of dicotyledonous angiosperm plants and described four different promoter types, which are likely to have replaced each other during evolution [5].

In this study we aimed at searching for widely conserved PEP-promoters in plastomes of the above mentioned taxa. By "widely conserved" we mean the cases when the regions upstream of orthologous genes across the high-level taxonomic divisions can be aligned. The promoters confined to only vascular plants or the red line lineages are not examined here (e.g., the NEP-promoter of gene *clpP *in vascular plants). In our analyses using the fixed consensus as a query produced massive under-predictions, or, alternatively, massive over-predictions, which suggests that querying without taking into account the alignment of 5'-leader regions is obviously misleading.

## Materials and methods

The regions of up to 1000 bp length upstream from all protein-coding genes (90 genes per species at average) in plastomes of species listed in Additional file 1 were extracted from GenBank, and multiple alignments of the regions were constructed. Searches of promoters were conducted using two original algorithms: the *first *to pre-select leader regions with candidate PEP-promoters (several candidates were found per region), and the *second *to build a multiple alignment keeping one of the candidate promoters in each of the regions. The alignment was constructed to reveal the two bacterial type boxes and cover the taxonomic diversity of the above mentioned lineages as wide as possible. In a *positive *prediction, the alignment of the boxes, linker and some flanking regions was required to have a good quality (see below). Otherwise, a *negative *prediction is produced and a PEP-promoter is not detected with our method. Evidently "positive prediction" means the prediction of a PEP-promoter and "negative prediction" means the lack of positive prediction. Notably, the positive predictions contained experimentally proved PEP-promoters and often their TG-extensions, which indicates that these are not false positives. Also, in all negative predictions the alignment had a considerably lower quality compared to the minimal quality among all positive predictions. All predicted PEP-promoters were located within approximately 40 bp-long highly conserved regions flanked by less conserved 3'-areas and highly variable 5'-areas.

The idea of the *first *algorithm. Given is a set of *n *leader regions. The goal is to find a subset of the set with one potential promoter in each region such that their total pair-wise similarity is maximal comparing to any other collection of potential promoters in that subset; the subset size is simultaneously maximized. In order to increase search speed, randomly selected regions are set as "linked" and the promoter similarity is estimated only within the linked pairs of regions. It formally means that we consider a graph with *n *vertices, each assigned a leader region, but only linked regions are connected by an edge in the graph. As a result, the complexity of comparing all pairs of candidate promoters to determine their total similarity is reduced in our algorithm by means of considering a large number of randomly defined sets of edges, i.e. randomly constructed graphs with *n *vertices assigned the same regions but connected by different edges. By doing so, the computing time becomes square to number *n *of the regions and cubic to their average length. The algorithm is designed for effective parallelization to enable mass processing of large amounts of long regions in feasible time. The enhanced performance of the parallel implementation allows to compute a solution closer to the maximum quality of the alignment. The algorithm is highly scalable and provides for the approximately linear growth of performance with the number of available processors up to 2000.

The idea of the *second *algorithm. Along a fixed phylogenetic species tree, the algorithm aligns leader regions with respect to one of the candidate promoters selected by the first algorithm, from the promoter start up to the start codon. It uses a common observation that promoters, as well as transcribed regions, can be well aligned, in contrast to the region upstream of the promoter. The algorithm takes a non-binary (which is often the case) species tree and during the run reduces it to a binary tree in a variety (or even all) possible ways. Each leaf of the tree bears an orthologous gene leader region from the corresponding species. The alignment is constructed as follows. First, each leaf is assigned a nucleotide frequency distribution at each position of the sequence: the distribution contains a unity for the observed nucleotide type and three zeros for the unobserved. A zero distribution contains four zeros. Then, at each inner node, *two *distribution sequences at its descendant nodes are aligned by any applicable algorithm, with an award for matching two distributions not pre-defined, but calculated anew at each position *j *taking into account the length of each descendant branch. The award is estimated as a scalar square of the difference between two nonzero distributions weighted for different nucleotide types. The penalty for inserting a gap symbol (i.e., for the alignment of zero and nonzero distributions) is a decreasing function of the number of contiguous gaps: the longer the gap region, the lower the penalty. Two zero distributions are forbidden to align. At each position of the alignment, the distribution in the ancestral sequence is a half-sum of the two distributions in the descendants. When the root distribution sequence is constructed, the algorithm projects the gaps along the tree to its leaves onto the extant sequences, thus obtaining the final multiple alignment. The complexity is linear to the number of leaves. Different binary tree resolutions are compared on the basis of the corresponding *alignment quality*, which is estimated as follows: , where *N*_*a *_is the number of totally conserved (containing the same character) single columns, *N*_*s *_- the number of totally conserved regions (two or more contiguous totally conserved columns, *l*_*i *_is the number of columns), *N*_*b *_- the number of "nearly" conserved columns (with one non-matching character); *b, c *and *s *are parameters. Computing an alignment of 16 sequences with the length of 120-223 bases requires less than one second on a 3 GHz Pentium-4 PC. The automatically computed alignments were manually checked and minor corrections were introduced if so required. Both algorithms are implemented as 32-bit command line utilities written in ANSI C, which can be compiled with many popular compilers and run under Windows or Linux. The algorithms and their detailed descriptions are available from [6,7].

Testing of the algorithms and their comparison with "common" local alignment algorithms (see the introduction and the list of references in [8]) are described in [9-11].

## Results

Table 1 contains the species from add. file 1 predicted to possess at least one *widely *conserved promoter in the plastome. Predictions are identical for their close relatives with a corresponding orthologous gene (not shown). Within flowering plants the promoter sequences are similar and well aligned, therefore we illustrate results on *Arabidopsis thaliana *and *Spinacia oleracea *only. The five positive predictions are described below. Our analyses suggest that *widely *conserved promoters are absent elsewhere in streptophyte plastomes.

**Table 1:** Estimated coordinates of the transcription initiation sites of the predicted PEP-promoters

Species	*psaA*	*psbA*	*psbB*	*psbE*	*rbcL*
*Arabidopsis thaliana*	Ex -188	Ex -77	-170	-125	-177
*Spinacia oleracea*	Ex -179	Ex -82	-175	-150	-176
*Cycas taitungensis*	Ex -156	-60	Ex -170	-141	-156
*Cryptomeria japonica*	Ex -142	-58	Ex -142	-137	-161
*Pinus koraiensis*	Ex -158	-52	-193	-148	-136
*Pinus thunbergii*	Ex -158	-52	-180	-145	-127
*Welwitschia mirabilis*	Ex -156	Ex -51	-271	-31	-136
*Adiantum capillus-veneris*	Ex -163	Ex -55	Ex -291	-191	-157
*Angiopteris evecta*	Ex -152	Ex -69	Ex -181	-142	-148
*Psilotum nudum*	Ex -147	Ex -53	Ex -178	-127	-140
*Huperzia lucidula*	Ex -153	Ex -55	Ex -187	-134	-150
*Anthoceros formosae*	Ex -155	=	=	-143	-160
*Aneura mirabilis*	Pseudo	Ex -54	Pseudo	Pseudo	-148
*Marchantia polymorpha*	Ex -149	Ex -53	=	-132	-124
*Physcomitrella patens*	Ex -161	Ex -53	=	-145	-143
*Chara vulgaris*	Ex -199	-121	Ex -179	=	-154
*Chaetosphaeridium globosum*	Ex -154	Ex -57	Ex -161	-119	-102
*Staurastrum punctulatum*	Ex -235	Ex -59	-190	-154	-219
*Zygnema circumcarinatum*	Ex -157	Ex -58	-159	-122	-168
*Chlorokybus atmophyticus*	=	=	-266	=	=
*Mesostigma viride*	=	Ex -53	-89	=	=
*Bigelowiella natans*	=	-136	=	=	=
*Cyanophora paradoxa*	=	-61	=	=	=

Coordinates are relative to the start codon. The "Ex" means the presence of the 5'-extension TG of the "-10" box, "Pseudo" marks a negative prediction for the pseudogene, "=" - a negative prediction for the functioning gene.

**Figure 1:**
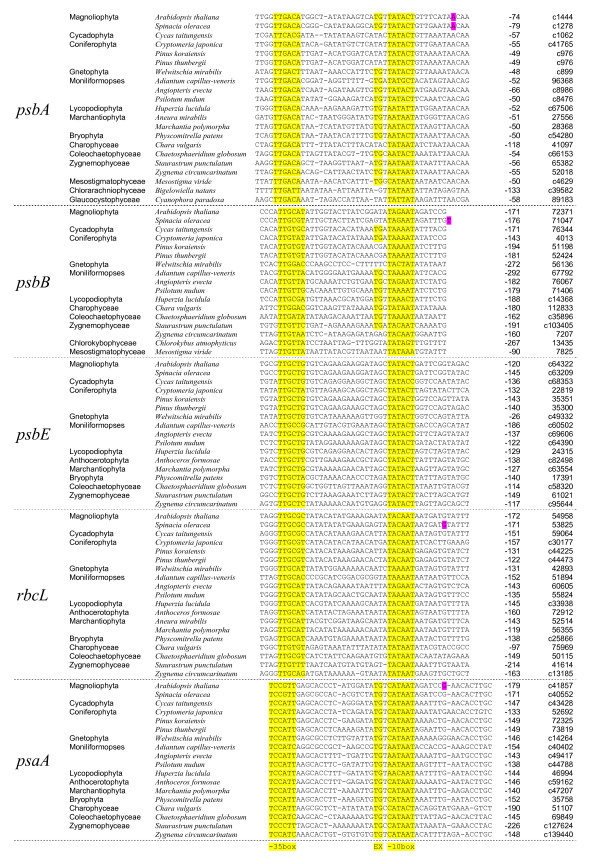
**Predicted promoters upstream of genes *psbA*, *psbB*, *psbE*, *rbcL*, *psaA***. In the cells of first column only first occurrences of each taxon name are given. In yellow are the promoter boxes and the 5'-extension of the "-10" box. Numbers are the distance to the start codon; its location is given in the last column, prepended with "c" for complement sequences. In violet are the experimentally identified transcription initiation sites in *Arabidopsis thaliana *and *Spinacia oleracea *upstream of *psbA*, *psbB*, *rbcL*, *psaA*.

**Figure 2:**
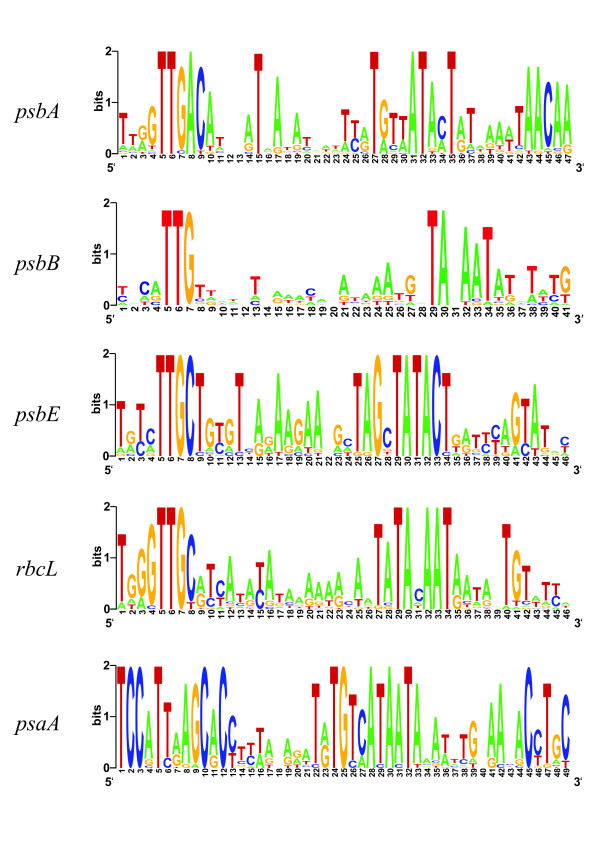
**Nucleotide frequency distribution for the alignments shown in Fig. 1**.

**Gene *psbA ***(protein D1 of the photosystem II active center) in plastomes. Promoters of this chloroplast gene were experimentally studied in selected species, including *Arabidopsis*, mustard, and spinach [3,12,13], for which our predictions are in good agreement with the experiment. The algorithm predicted candidate conserved promoters upstream of this gene in most Streptophyta, primary and secondary endosymbionts, *Bigelowiella natans *from the Chlorarachniophyceae, and *Cyanophora paradoxa *from the Glaucocystophyceae (ref. to Fig. 1, *psbA*). The gene alignments are given in Fig. 1, per-site nucleotide frequency distributions are given in Fig. 2 (constructed with the Weblogo program [14]). We suggest that this ancient promoter with the consensus TTGACA-15-TGTwATAmT is ancestral for at least all Streptophyta. The linker between the boxes is usually 18 bases long, but is 17 bases in *Cycas taitungensis*, *Adiantum capillus-veneris*, *Staurastrum punctulatum*, *Mesostigma viride *and *B. natans*. Many predictions possess the 5'-extension (TG or TGTG) of the "-10" box, which enhances the promoter efficiency. In the gymnosperm *C. taitungensis*, the predicted "-35" box essentially differs from the alignment consensus and the bacterial-like promoter. The *psbA *promoter was not found in the hornworts *Anthoceros formosae*, although in other bryophytes it is highly conserved. In the early emerging alga *Chlorokybus atmophyticus *only the "-35" box was identified, while the complete promoter was found in *M. viride*. Two dodder species (*Cuscuta gronovii*, *C. obtusiflora*) with a largely reduced plastome also lack the *psbA *promoter, which, however is found in their close relatives (*C. exaltata*, *C. reflexa*) and most angiosperm plants. The lack of promoters correlates with the reduction of genomes: *Cuscuta gronovii *and *C. obtusiflora *do not photosynthesize and lack most of the photosynthetic genes. Although the *psbA *gene retains an open reading frame, it lacks the PEP-promoter and is probably poorly expressed compared to photosynthetic species.

**Figure 3:**
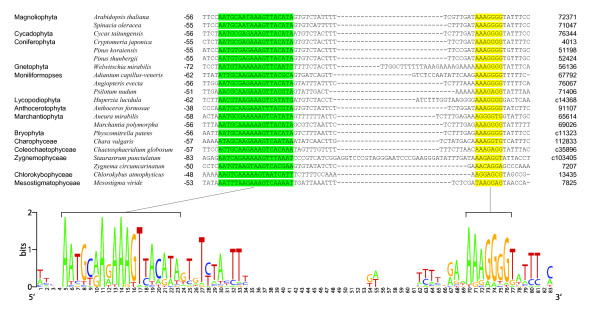
**The 5'-leader regions upstream of gene *psbB***. In the cells of first column only first occurrences of each taxon name are given. Numbers to the left of the sequences are distances from the 5'-edge to the start codon, which location is specified in the last column ("c" stands for complement sequences). In spinach the region is located precisely between the mRNA cleavage site and the start codon. Conserved putative mRNA-protein binding sites downstream of the cleavage site are shown in green. Conserved putative ribosome binding sites close to the start codon are in yellow.

**Gene *psbB ***(a chlorophyll apoprotein of photosystem II CP47) in plastomes of Streptophyta. For this gene, the transcription start is experimentally identified in spinach (*S. oleracea*) [15]; it adjoins the 3'-end of the accordingly named sequence in Fig. 1, *psbB*. A conserved promoter is predicted in most vascular plants: in angiosperms (*A. thaliana*, *S. oleracea*), gymnosperms (*Cycas taitungensis*, *Cryptomeria japonica*, *Welwitschia mirabilis*, *Pinus *spp.) and pteridophytes (*Adiantum capillus-veneris*, *Angiopteris evecta*, *Psilotum nudum*, *Huperzia lucidula*). A related promoter is predicted in some algae (*Chaetosphaeridium globosum*, *Chara vulgaris*, *Staurastrum punctulatum*, *Zygnema circumcarinatum*, *Chlorokybus atmophyticus*, *Mesostigma viride*), ref. to Fig. 1, *psbB*. This promoter is highly conserved in *C. taitungensis*, *C. japonica*, pteridophytes and streptophyte algae *C. globosum*, *C. vulgaris*, *S. punctulatum*, and less conserved in *Z. circumcarinatum*, *C. atmophyticus *and *M. viride*. It possesses the "-10" box TG-extension. In the early branching *C. atmophyticus *and *M. viride*, several potential promoters are predicted in 5'-leader regions; however these cannot be unambiguously added to the alignment of Streptophytina (Fig. 1, *psbB*), especially in the regions between the boxes and start codons. Therefore, the promoters closest to the start codon are selected and shown for *C. atmophyticus *and *M. viride*. In bryophytes (*Aneura mirabilis*, *Anthoceros formosae*, *Marchantia polymorpha*, *Physcomitrella patens*), a conserved promoter was not found. Notably, the *psbB *sequence of *A. mirabilis *is annotated as a pseudogene in NCBI GenBank. The usual linker of 18 bp between the boxes is reduced to 17 bp in *W. mirabilis *and some algae (*C. atmophyticus*, *S. punctulatum*, *Z. circumcarinatum*). In the pines *Pinus koraiensis *and *P. thunbergii*, the sequence differences are not shown (they occur in between the end of the sequence in Fig. 1, *psbB *and the conserved processing site shown in Fig. 3).

**Gene *psbE ***(photosystem II cytochrome b559 protein alpha subunit) in plastomes of Streptophyta. Promoters were predicted in most land plants and the algae *Chaetosphaeridium globosum*, *Staurastrum punctulatum*, *Zygnema circumcarinatum*, ref. to Fig. 1, *psbE*. Negative predictions were obtained for the algae *Chara vulgaris*, *Chlorokybus atmophyticus *and *Mesostigma viride*, even though the region is conserved in their closer relatives. This gene is a pseudogene in the *Aneura mirabilis *plastome.

**Gene *rbcL ***(the large subunit of ribulose-1,5-bisphosphate carboxylase) in plastomes of Streptophyta. The promoter was experimentally characterized in spinach (*S. oleracea*) [13], and mustard (*Sinapis alba*) [12]. It was predicted in all land plants and in the streptophyte algae *Chaetosphaeridium globosum*, *Chara vulgaris*, *Staurastrum punctulatum*, *Zygnema circumcarinatum*, ref. to Fig. 1, *rbcL*.

**Gene *psaA ***(apoprotein A1 of photosystem I P700) in plastomes of Streptophyta. Promoter and the transcription initiation site for this gene were experimentally characterized in *Arabidopsis thaliana *[16]. In *Aneura mirabilis *it is a pseudogene. The promoter was predicted in almost all land plants and streptophyte algae, except for *Chlorokybus atmophyticus *and *Mesostigma viride*, see Fig. 1, *psaA*. This promoter differs from all other predictions and the bacterial σ-70 promoter. Its "-10" box consensus is CATAAT, which differs from the bacterial type at the first position. At the 5'-end of the box a conserved putative extension is found with the consensus TrTGT. The predicted "-35" box is even more divergent from its counterparts, despite being located within a long conserved region.

Although the alignments shown Fig. 1 are unambiguous within the lineages, neither can be extended onto the Euglenozoa, Chlorophyta, Rhodophyta, Cryptophyta, diatom and other algae with plastids similar to those of the Rhodophyta, see add. file 1.

Normally, the entire promoter region, not only the boxes, is more conserved comparing to the rest of the leader region, which hampers distinguishing between regulated and non-regulated promoters.

We illustrate the comparison between wide and local conservations on the PEP-promoters of genes *ycf1*, *rps4 *and *psaJ*. The promoters were experimentally identified in *Arabidopsis thaliana*. These genes are among the 85 protein-coding genes in the plastome of *A. thaliana*. They are not widely conserved.

The *ycf1 *gene encodes an unknown function protein and has PEP-promoter *ycf1*-34 with a smaller distance between the "-35" and "-10" boxes than normally [3]. This promoter overlaps with NEP-promoter *ycf1*-39. PEP-promoters very similar to *ycf1*-34 with unambiguous multiple alignments of the 5'-UTR regions are found in most eudicotyledonous, magnoliid and basal magnoliophyte plants. Some species (including *Cucumis sativus*) possess a much longer 5'-UTR region, while in others (including *Ranunculus macranthus*) the *ycf1 *PEP-promoter is not found. In monocotyledonous (Liliopsida), gymnosperm and pteridophyte plants possessing the *ycf1 *gene, its putative PEP-promoters are found but differ considerably from those in eudicotyledons, magnoliids and the basal Magnoliophyta. The promoter in *A. thaliana *is most similar to that from the cycadophyte *Cycas taitungensis*.

In *A. thaliana *the gene *rps4 *encoding ribosomal protein S4 has PEP-promotor *rps4*-123 [3]. Similar promoters with unambiguous 5'-UTR multiple alignment are found only in selected species of Brassicaceae: *Arabis hirsuta*, *Barbarea verna*, *Crucihimalaya wallichii*, *Draba nemorosa*, *Lepidium virginicum*, *Lobularia maritima*, *Nasturtium officinale *and *Olimarabidopsis pumila*. The plastomes of *B. verna*, *D. nemorosa*, *L. maritima *and *O. pumila *contain single nucleotide insertions in between the boxes;*Arabis hirsute *has a single nucleotide deletion. The promoter region is variable even across close species (*Aethionema cordifolium*, *A. grandiflorum*, *Carica papaya*, *Citrus sinensis*) but their 5'-UTR regions can still be well aligned.

*A. thaliana *was experimentally found to possess a Sig2-dependent promoter upstream of gene *psaJ *encoding photosystem I active center subunit IX, with a 37 nucleotide-long 5'-UTR [17]. Although well aligned across all eurosids II, its 5'-UTR regions are conserved only within Brassicaceae and diverge already in *C. papaya*.

## Discussion

Conserved promoters are found in the monophyletic Streptophyta and in two distant species, *B. natans *and *C. paradoxa*. Notably, even though *B. natans *belongs to the Cercozoa, its plastome is similar to that of green algae [18]. On the contrary, the plastome of *C. paradoxa *is different in many respects [19,20].

There are many reasons why PEP-promoters upstream of the protein-coding plastome genes are scarce. Their loss may be related to the evolutionary changes of sigma subunit paralogs and phage-type RNA polymerases that lead to rapid replacements of the PEP-promoter. Indeed, the PEP sigma subunits vary already between maize, poplar and thale cress: e.g., maize possesses two Sig2 paralogs and lacks Sig4, while in poplar *sig4 *is a pseudogene, and thale cress possesses a Sig4 and only one Sig2, [21]. Also, promoters can be lost with their nuclear sigma subunit-encoding genes, such as the Sig4-dependent *ndhF *promoter in poplar [5]. Some dicotyledonous plants, including *Arabidopsis *and *Nicotiana*, have gained the additional phage-type RNA polymerase RpoTmp, which is active in chloroplasts and mitochondria of these plants but is missing from monocotyledonous plants (unpublished dissertation by K. Kühn, 2006). Only one phage-type RNA polymerase, RpoTp, is known from plastids of monocots (*Zea*, *Triticum*), two phage-type RNA polymerases - from plastids of dicots (*Arabidopsis*, *Nicotiana*): RpoTp in chloroplasts and RpoTmp in both chloroplasts and mitochondria. The moss *Physcomitrella patens *also has two phage-type polymerases, RpoT1 and RpoT2, which target both chloroplasts and mitochondria [22]. Promoters can emerge *de novo*, as has been shown, e.g., for the *ndhF *promoter [5]. Others are lost together with plastome genes, e.g., the *chlL *promoter in flowering and some other plants (according to the GenBank records). Another possible factor in rapid promoter turnover in plastids may be tissue-specific differentiation of plastid types, especially in vascular and, particularly, flowering plants, which evolved a rich diversity of sigma subunits [21] and phage type RNA polymerases. Often the promoter boxes are functionally substituted by the transcription activation factor binding sites [4].

In parasitic, non-photosynthesizing plants, such as dicotyledonous dodder (*Cuscuta *spp.) and liverwort *Aneura mirabilis*, many chloroplast genes are pseudogenes [23] and promoters of these genes are lost too. The promoter conservation might become lower in the presence of alternative promoters. The promoter might have undergone rapid evolution [3,5] and become unrecognizable. It also might be located beyond the 1000 bp distance from the start codon and thus be overlooked in our analyses.

Given these multiple reasons to expect fast evolution and rapid turnover of the chloroplast promoters, one may ask why some of them, such as the five promoters described above, are so widely conserved? One possible explanation is that three of the conserved promoters regulate the expression of the photosystem components and that the stability of the promoter structure is important to maintain high expression of genes *psbA*, *psbB*, *psaA*; due to the light-dependent translation regulation of *psbA*, a high amount of mRNA is built up in the dark and translated under light [24]. Conserved promoters upstream of *psbA *and *psaA *may also be required to form polycistronic mRNAs, which encode, along with the photosystem components, tRNA and proteins involved in translation that also have to be expressed at high levels: *psbA *appears to belong to the same operon as histidine tRNA, while *psaAB *and *rps14 *are in an operon with methionine tRNA. The *psbEFLJ *operon and *psbBTHpetBD *operon might be formed likewise. The other conserved promoter regulates *rbcL*, the large subunit of a key enzyme involved in the carbon dioxide fixation during the Calvin cycle, the most abundant enzyme in the biosphere, whose gene also must be highly expressed. When a gene is highly transcribed and regulated by a single promoter, the selection pressure prevents any considerable change in the promoter's structure to provide for its effective binding to the polymerase.

Relatively lower conservation of the PEP-promoters of housekeeping genes (viz., tRNA, rRNA, ribosomal protein and PEP subunit-encoding genes, etc.) might be explained by the presence of NEP transcription: e.g., the *rpoB *transcription is entirely NEP-mediated, although most genes possess both PEP and NEP-promoters. This is the case of the *ycf1 *and *clpP *genes, which were experimentally shown in *Arabidopsis thaliana *to be under several promoters recognized by PEP with different subunits and two NEP, RpoTp and RpoTmp, [22].

Operonic organization and RNA polymerase competition are important factors explaining the effect of genome rearrangements on the evolution of promoters. Thus, the loss of the common *ndhF *promoter and the emergence of a new one upstream of gene *ndhF *in poplar (*Populus alba*, *P. trichocarpa*) concur with the deletion of a neighboring gene [5].

Some conserved promoters might be overlooked. For instance, the well studied *psbC *promoter is located within a coding region of other gene (according to the GenBank records) and its conservation cannot be assessed without estimating the synonymous vs. non-synonymous substitutions ratio, which is yet to be incorporated in our approach. Similar promoter-like regions were observed within other coding areas (unpublished data), but their role awaits explanation.

## Competing interests

The authors declare that they have no competing interests.

## Authors' contributions

VAL and AVS performed the analyses, interpreted the results and developed the algorithms. LIR programmed the algorithms and ran computations. All authors contributed equally to preparing the manuscript.

## Reviewers' comments

Reviewer's report 1

Arcady Mushegian, Stowers Institute

The manuscript by Lyubetsky et al. examines the conservation of promoters in the choroplast genes of Streptophyta. The evidence is presented that, across large evolutionary distances (i.e., larger than the flowering plants clade) only a handful of promoter sequences contains conserved regions. This is an interesting observation suitable for publication in the Discovery Notes section of Biology Direct.

1) 1st paragraph: the authors assert that there is no published evidence on searching for promoters at the genome scale. This is not true and needs to be qualified: there are many papers about eukaryotes and several about either methods to detect or databases of detected promotors in various groups of bacteria, some of which have been obtained using intergenomic conservation as one of the criteria. Citing the research behind J.Collado-Vides databases or RegulonDB might be in order.

Response: This sentence lacks the word "..plastid.." which occurs widely in our text and is present in the title. We now refer to the works by professor Collado-Vides [2], which contain references to databases on promoters and regulation factors including the RegulonDB database. These databases and other citations in [2] are related to selected gamma-, alpha-proteobacteria and eukaryotic nucleoms. We do not see them as directly related to the "searching for the plastid promoters at the genomic scale". Particularly, the RegulonDB database does not contain photosynthesis and many other plastome genes because they lack in *E. coli*. The intergenomic conservation ideology is used in our algorithms [6,7] but in a form different from that in [2].

2) Methods: references 4 and 5 are links to the authors' website with the documentation of their software. Why the reliance on the original code instead of the established methods of motif search and sequence alignment? Please explain crucial differences in the algorithms and how the homegrown ones were tested.

Response: Studies [9,10] report testing of the "first" algorithm in our approach in the comparison with established local alignment algorithms. The "second" algorithm and its testing was reported during a conference [11]. Widely used "standard" programs did not produce better promoter predictions (they are described in [8] and many related references). An explanation might be that we define a PEP-promoter as two boxes separated by a region (sometimes with a TG extension) variable in terms of structure and length; the imposed requirements are the degree of the variability of this region, the linker between the "-10" box and the start codon and the 5'-end of the "-35" box. The alignment of leader regions was built based on the precomputed two-boxed structures. It is more efficient to build it along a (usually known) species tree and not construct the alignment and the tree anew together as some approaches do. Ideologically the algorithms are described in the text, full details are given in [6,7] and demonstrate their different performance comparing to other published methods.

3) A suggestion that may help to provide a more complete picture of the evolutionary trends in chloroplast promoter conservation: *A. thaliana *chloroplast has 85 protein-coding genes. Can we have a table that shows, for each gene, how broadly its promoter is conserved?

Response: The "Results" section now contains an analysis of PEP-promoter conservation upstream some coding genes in *A. thaliana*. An analysis of all 85 genes would be a subject for a separate publication. We show (as also noted in [5]) a typical problem in finding non-widely conserved promoters. Thus, well studied gene *ndhF *in *A. thaliana *is found to have only one PEP-promoter out of the four types known in Magnoliophyta, which is conserved across the Brassicaceae and predicted in all sequenced eurosids II and in *Vitis vinifera *[5]. Chloroplast PEP-promoters are experimentally unidentified for many coding genes in *A. thaliana*, while for many they are [3]. These promoters are conserved also in the Brassicaceae but already in eurosids II their recognition depends on imposed cut-offs and requires biological validation. For widely conserved promoters over-prediction is much lower than for promoters conserved within a thin lineage where the leader regions did not diverge to a noticeable extent.

## Reviewer's report 2

Alexander Bolshoy, University of Haifa (nominated by Purificación López-García, Université Paris-Sud)

In the paper of Lyubetsky et al. conservation and variability of the plastid promoters is studied, and, to the best of my knowledge, for the first time at the whole genome level. Undoubtedly, the problem is important and non-trivial. The authors obtained unexpected result: promoter regions in plastids are less conservative than corresponding coding sequences. To identify promoters the authors proposed an original method of searching short motifs surrounded by certain other motifs. Thus, the proposed article includes an interesting problem, original methods to solve it and non-trivial results of analysis of promoter regions. It makes this article suitable for publication in the Discovery Notes section of Biology Direct.

My remarks:

1) In Background section you use a term "lower conservation". Can you show how have you compared protein conservation with promoter conservation? Response: Comparing to the PEP-promoters, their regulated proteins are always widely conserved and well aligned. A family present in vascular pants is almost ubiquitous, while known widely conserved PEP-promoters are only five. PEP-promoters might be more abundant than NEP-promoters: the knockout of RpoTp-NEP is not lethal for *A. thaliana*, while the PEP-promoter loss (e.g. in *Epifagus virginiana*) entails the loss of numerous genes. The authors are unaware of detailed estimates.

2) In Background section you use the term "widely" to indicate that the leader region sequences upstream orthologous genes can be aligned across high-level taxonomic divisions. Please, give some details for better understanding of the term "widely conserved"?

Please refer to Response #3 to Yu.W.

3) In Background section the following phrase "... using the fixed consensus as a query produced massive under-predictions, or, alternatively, massive over-predictions..." needs some explanation.

Response: A simple approach to the promoter search is to define a conserved query mask. Using masks very close to, e.g., the bacterial sigma-70 consensus, will lead to under-predictions because reliable PEP-promoters of different structure will be overlooked. Using diverged masks will lead to numerous false predictions. We believe that using a fixed per-site nucleotide frequency queries is not a perspective.

4) Materials and methods. Please, give a short description of your algorithms.

Response: We developed an original approach to the promoters search. At the first stage we find a two-boxed signal via local multiple alignment (the first algorithm, ref. to Response to A.M #2). For each leader region the algorithm predicts a number of candidate "-35" and "-10" boxes. The second algorithm aligns the promoter region, about 20 nucleotides upstream its "-35" box and the transcribed region up to the start codon (the part of the alignment is given in Fig. 1) and chooses the putative boxes taking into account the distance between them (typically 17-18 nucleotides) and their affinity on the species tree (closer species have more similar sequences). The algorithms are described in detail in [6,7].

5) Results. Why the authors insist to strengthen differences between plastid REP-promoter of *psaA *gene and bacterial σ-70 promoters?

Response: The *psaA *leader regions have a reliable long alignment, which accents the fact that this promoter considerably differs from the bacterial sigma-70 consensus.

## Reviewer's report 3

Yuri I. Wolf, National Center for Biotechnology Information (nominated by Purificación López-García, Université Paris-Sud)

The authors report the virtual lack of conservation of Plastid-Encoded Polymerase promoters among the various lineages of plants. The finding is quite noteworthy and would be of interest to those who study the evolution of regulatory elements and plastid genomes.

1) p. 2. "Plastid genes and their promoters are believed to be evolutionarily conserved across large taxonomic lineages". This is a strong statement that requires at least a couple of references, indicated who, when and in what form expressed these beliefs.

Response: In [2, 9.7c] (this reference is added) the authors state that "The structure of chloroplast... genes is widely conserved across lineages. Their evolutionary rate is much lower than that of nuclear genes." This seems to be a common knowledge from textbooks (references can be added if necessary). Our logic was first straight: highly conserved genes cannot have low conserved promoters. But out results show the opposite. The phrase "and their promoters" is now removed.

2) p.3 and throughout. "The term "widely" is used to indicate...". The authors attempt to clarify the usage of the term "widely", but actually just substitute it by no less vague "across high-level taxonomic divisions". I suggest to specify the "high-level taxonomic divisions" used in the definition of "widely" and avoid the italicized usage of this term further in the text.

Response: An alignment was called "widely conserved" when included the Magnoliophyta and at least two representatives (at least one must not be a vascular plant) from Cycadophyta, Coniferophyta, Gnetophyta, Moniliformopses, Lycopodiophyta, Marchantiophyta, Bryophyta, Charophyceae, Coleochaetophyceae, Zygnemophyceae, Mesostigmatophyceae, Chlorarachniophyceae or Glaucocystophyceae. Each high lineage from Fig. 1 is represented by few species because other species can usually be unambiguously aligned. These lineages are unbalanced in terms of molecular taxon sampling and are here represented by similar numbers of species. The term "widely conserved" will hopefully be given a more precise definition in the future.

3) pp. 4-6. The gene-specific section of the Results reads like a verbal narration of the content of the Table 1. It is not clear why the authors need such a detailed listing of facts that don't seem to lead to any particular conclusions. I would recommend considering the possibility of removing this part from Results altogether, joining Results and Discussion and use the extra available space to somewhat expand the Methods section.

Response: The "Results" do not just state the fact of the widely conserved promoter and its distance from the gene (which is indeed evident from Table 1) but also comparisons of the orthologous gene promoters supported by the alignment analyses and interpretations of published data. The authors believe this section should be kept at least structurally. It might be technically merged with the Discussion but its contents should remain. Discussion elements in the Results are directly related to the details described. If the note is to be reduced, we argue for moving Fig. 2 (and, if needed, Table 1) into the supplementary data.

4) Promoter blocks for different genes seem to be aligned, but all shown sequences have different lengths. This leads to a seemingly paradoxical result - the magenta mark for the experimentally identified transcription initiation site in *psbB *of *Spinacia oleracea *highlights an empty space.

Response: The Figure 1 shows a good alignment, which length cannot be amended. If the *psbB *alignment is appended some columns to the right, its quality will decrease. In magenta is now a character existing in this position in spinach an experimentally proved to be at the transcription start.

## Supplementary Material

Additional file 1 **The list of plastomes examined for conserved PEP promoters**. The data were extracted from GenBank, NCBI. Click here for file
